# A grounded theory of the implementation of medical orders by clinical nurses

**DOI:** 10.1186/s12912-024-01775-6

**Published:** 2024-02-13

**Authors:** Monireh Asadi, Fazlollah Ahmadi, Easa Mohammadi, Mojtaba Vaismoradi

**Affiliations:** 1 Nursing Department, Faculty of Medical Sciences, Tarbiat Modares University, P.O. Box. 14155-4838, Tehran, Iran; 2 Faculty of Nursing and Health Sciences, Nord University, Bodø, Norway; 3 Faculty of Science and Health, Charles Sturt University, Orange, NSW Australia

**Keywords:** Grounded theory, Medical orders, Nurses, Nursing documentation, Patient safety

## Abstract

**Objective:**

To explore the process of implementing medical orders by clinical nurses, and identify specific areas of concern in the implementation process, and uncover strategies to address these concerns.

**Background:**

The implementation of medical orders is a crucial responsibility for clinical nurses, as they bear legal accountability for the precise implementation of directives issued by medical practitioners. The accurate implementation of these orders not only shapes the quality and safety of healthcare services but also presents numerous challenges that demand careful consideration.

**Method:**

This study employed a qualitative design using a grounded theory approach to construct a comprehensive theoretical framework grounded in the insights and experiences of nurses operating within the hospital settings of Iran. The study encompassed 20 participants, comprising 16 clinical nurses, two nurse managers, and two specialist doctors working in hospital settings. The selection process involved purposeful and theoretical sampling methods to ensure diverse perspectives. Data collection unfolded through in-depth, individual, semi-structured interviews, persisting until data saturation was achieved. The analytical framework proposed by Corbin and Strauss (2015) guided the process, leading to the development of a coherent theory encapsulating the essence of the study phenomenon.

**Findings:**

The primary finding of the study underscores the significance of ‘legal threat and job prestige’ highlighting diverse repercussions in case of errors in the implementation of medical orders. At the core of the investigation, the central variable and the theory of the study was the ‘selective and tasteful implementation of orders to avoid legal and organizational accountability.’ This indicated a set of strategies employed by the nurses in the implementation of medical orders, encapsulated through three fundamental concepts: ‘accuracy in controlling medical orders,’ ‘untruth documentation,’ and ‘concealment of events. The formidable influence of legal threats and job prestige was further compounded by factors such as heavy workloads, the doctor’s non-compliance with legal instructions for giving verbal orders, the addition of orders by the doctor without informing nurses, and pressure by nursing managers to complete documentation. The resultant psychological distress experienced by nurses not only jeopardized patient safety but also underscored the intricate interplay between legal implications and professional standing within the healthcare framework.

**Conclusion:**

Alleviating staff shortages, enhancing the professional rapport between doctors and nurses, offering legal support to nursing staff, implementing measures such as recording departmental phone conversations to deter the non-acceptance of verbal orders, fostering an organizational culture that embraces nurse fallibility and encourages improvement, and upgrading equipment can ameliorate nurses’ apprehensions and contribute to the safe implementation of medical orders.

**Supplementary Information:**

The online version contains supplementary material available at 10.1186/s12912-024-01775-6.

## Introduction

Nurses dedicate a significant portion of their daily work schedule to the implementation of medication orders. This is a multi-step and complex process and is vital for patient’s health [1].

Nurses play a pivotal role in carrying out medical orders [1–3] and bear responsibility and accountability for their accurate implementation [2, 4].

The process includes stages such as checking medical orders, prescribing medications, and documenting executed orders. Ensuring the proper implementation of medical orders by nurses is essential for ensuring patient safety [5, 6].

Maintaining patient safety relies significantly on clear and carefully reviewed medical orders by nurses, serving as mechanisms to prevent practice errors [7]. However, the process of implementing medical orders is prone to errors, with individual and environmental factors, including work conditions and personal and organizational reasons, adding complexity and challenges to the process. Enhancing the quality of this process depends on identifying and addressing these challenges [2].

Incorrect implementation of medical orders, particularly in medication administration, poses a significant risk of severe harm to patients [4]. This can lead to prolonged hospitalization [8], increased healthcare costs, and legal repercussions [9]. Shortages of nurses, job-related stress, and the workplace atmosphere contribute to errors in implementing medication orders [10]. Initiatives by nurse managers to enhance workplace conditions positively impact the implementation of orders by nurses [11]. Additionally, creating an environment where nurses can report errors in the implementation of medical orders without fear of consequences is crucial [10].

The British International Association of Patient Safety emphasizes that re-checking or double-checking orders before implementation by nurses is a preventive measure against errors [12]. However, nurses often find it challenging to perform these checks, particularly in overcrowded wards [13]. Unclear medical orders contribute to interruptions or errors during implementation [14]. The frequent alteration of orders is a source of concern for nurses [1]. Additionally, the implementation of unclear verbal orders creates confusion for nurses [15, 16]. Nurses frequently express dissatisfaction with doctors’ unclear or verbal instructions, highlighting that vague medical orders signify a lack of professional communication between the doctor and the nurse [17]. Inadequate professional communication between nurses and physicians stands as a primary cause of medical errors [17–19].

A mutual understanding between the nurse and the doctor is crucial for discussing the patient’s condition and improving the quality of care [17]. Effective planning of patient care relies on communication through written orders between the doctor and the nurse. Consequently, the implementation of medical orders is documented, allowing for the systematic monitoring of the therapeutic process [16, 20]. Nevertheless, non-functional policies and organizational rules in the implementation process can lead nurses to deviate from established rules and standards [13].

Identifying underlying factors influencing errors in the implementation of medical orders can be gleaned from the lived experiences of nurses [21]. Recognizing the critical significance of accurate medical order implementation by nurses and its direct impact on the quality and safety of patient care, it is essential to explore nurses’ interactions with medical orders and elucidate the implementation process. Therefore, this study aimed to explore the process of implementing medical orders by clinical nurses, and identify specific areas of concern in the implementation process, and uncover strategies to address these concerns. Identifying specific areas of concern in the implementation process and uncovering strategies to address these concerns, this research offers insights that can enhance global practices in healthcare delivery and patient safety and pave the way for innovative approaches and improvements in the implementation of medical orders.

## Methods

From May 2021 to September 2022, this qualitative research employing a grounded theory approach was conducted. This approach facilitates the assessment of the nature, structure, process, and determining factors of exposure to social phenomena within their natural context [22]. The article adheres to the consolidated criteria for reporting qualitative research (COREQ) [23].

### Participants and setting

The study involved a participant group of 20 individuals, comprising 16 nurses, 2 nursing managers, and 2 medical doctors. Among them, there were 7 men and 13 women, and the average work experience was 12 years. Participants were recruited from a diverse pool, spanning 16 hospitals across three cities in Iran (Table 1).

**Table 1:** The demographic characteristics of the participants

Demographic variable	Chracteristics
Gender	Female #13 Male # 7
Education level	Bachelor’s degree #13 Master’s degree # 5 Medical doctor # 2
Range of work experiences (years)	Clinical nurse: 3–22 Nurse manager: 4–5 Medical doctor: 6–12
Workplace	Critical care unit # 5 Internal medicine # 4 Surgery # 6 Emergency # 5

Nurses were selected based on specific criteria, including holding a bachelor’s degree in nursing and possessing a minimum of one year of experience in implementing medical orders. A shared criterion for all participants was their expressed willingness to openly share their experiences.

The sampling process began purposefully and progressed through theoretical sampling until theoretical saturation was attained. Participants were selected based on information provided by preceding participants, continuing until collecting additional data yielded no further new or relevant findings. This approach ensured comprehensive exploration and understanding of the topic based on the depth and richness of participants’ experiences.

### Data collection

After securing permissions for the study, the principal researcher, a doctoral student, initiated contact with potential participants. Providing an introduction and explaining the research’s purpose, the researcher addressed queries, invited them for interviews, and, upon agreement, had participants sign an informed consent form. Convenient dates and times for interviews were determined. The main researcher conducted data collection through semi-structured, in-depth, individual phone interviews, adhering to COVID-19 health protocols. Due to the COVID-19 pandemic and the imperative to adhere to health and quarantine protocols, the researcher was unable to physically visit the departments. Additionally, given the heightened workload of nurses during the pandemic, it was not feasible to conduct interviews during their work shifts. Consequently, the researcher opted to call participants before the interview, conducting the interviews remotely over the phone.

Demographic data, including gender, education level, and nursing work experience, were also recorded. An open-ended question was employed at the beginning of interviews to encourage participants to freely share their experiences: ‘How do you implement medical orders in your everyday practice in the ward?’ Probing questions were asked based on the responses provided by the interviewees: ‘What do you mean?’, ‘Can you please explain it more?’, and ‘What was the result of this action?’.

Subsequent interviews were organized to address gaps in the emerging theory, using data gathered from previous interviews. The formulation of interview questions was grounded in theoretical sampling, with key informants chosen to ensure maximum diversity across gender, education level, service department type, and work experience until data saturation was achieved. It was deemed essential to conduct interviews with physicians and nurse managers to address data gaps regarding their roles and support in the process of implementing medical orders by nurses. Theoretical questions, formulated based on memos derived from interview data, were posed to elucidate the characteristics and dimensions of the study phenomenon. This iterative approach aimed to refine and enrich the evolving theoretical framework. Examples of questions were: ‘How do you receive, implement, and document telephone orders?’, ‘What new orders were added by the doctor to previously checked ones?’, ‘How do you manage them?’, ‘Did you have any experience with not accepting the nurse’s documented phone order from the doctor?’, ‘How did you deal with it and what was the result?’ (Supplementary file 1).

All interviews were recorded with participants’ permission. Each participant was interviewed once, and data saturation was achieved after 20 interviews. However, two additional interviews were conducted to ensure comprehensive data sufficiency. The researcher opted for these supplementary interviews as a precautionary measure. This deliberate approach aimed to affirm that the study had exhaustively explored the experiences and perspectives of participants, providing a robust foundation for analysis and interpretation. The interview duration ranged from 40 to 80 min, with an average of 50 min.

### Data analysis

The data collection and analysis occurred concurrently, with each subsequent interview informed by data collected and analyzed from the previous one. This iterative process facilitated data collection based on emerging concepts. The analysis method employed was the Corbin and Strauss (2015) approach to give meaning to the data and derive concepts related to the overall dataset. It involved the following dimensions:

- Open and substantive coding.
- Development of subcategories and then categories as concepts based on their characteristics and dimensions.
- Data analysis for context.
- Identifying the process.
- Integrating categories to develop themes.
- Presentation of the storyline and theory [24].

The interviews were transcribed verbatim and read multiple times to comprehend the participants’ statements comprehensively. During the transcription of interviews, meticulous attention was given to capturing every nuanced detail in the data. Semantic units in the data were identified, and corresponding codes were assigned. Through constant comparison of similarities and differences, codes were organized and grouped into subcategories. The data were then conceptualized, and categories were developed based on the researcher’s interpretation of the data. Memos, written during the interviews, guided theoretical sampling and facilitated the exploration of different and unexplored aspects of the phenomenon and the process. This approach contributed to the evolution and formation of themes as theoretical concepts and the overarching theory. An illustrative example of the data analysis is presented in Table 2. As to address rigor, the researchers’ sustained engagement with both the data and participants over an extended period contributed to the enhanced credibility of the findings [25]. To validate interpretations, member checking involved providing selected participants with a concise report of the findings for alignment with their experiences. Additionally, the entire process of data analysis, abstraction, coding, and the development of concepts and categories underwent scrutiny and approval by three nurse faculty members recognized as experts in qualitative research. Emphasis was placed on ensuring the applicability of the findings, aiming to extract operational strategies from participants’ perspectives and identify both obstacles to and facilitators of implementing medical orders.

**Table 2:** The process of developing the concept of unprofessional documentation

Meaning Units	Substantive code	Category	Theme
“I gave the patient’s gavage on a wrong volume, but I did not document it, because I would have been reprimanded.” (Participant 4, master’s degree, clinical nurse) “If I talk about the error in the implementation, it will be a legal headache, so I will not inform anyone.” (P20. master’s degree, clinical nurse) “I gave the wrong medicine to the patient and told the supervisor; he would inform everyone and raise my eyebrows. After that, I did not mention any of the errors in the implementation of orders.” (P19, master’s degree, clinical nurse). “The depressed patient died while we wrote in the report that the orders were implemented, and the patient was fine. The patient’s mother complained, we all destroyed the previous reports together and wrote the patient’s reports from the moment of arrival in such a way that the patient was in a bad mood and did not take medications, because of those new reports we were all acquitted.” (P18, master’s degree, clinical nurse). “The head of the department said that it was important that I write my report completely and according to instructions so that there was no legal trouble, and it did not matter if the work of the patient was not done, we also had to write unrealistic sometimes.” (13, master’s degree, clinical nurse). “Due to being busy, I often implement medical orders and my colleagues write the report or vice versa.” (P7, master’s degree, clinical nurse). “Often, after checking the doctor’s orders, I write the report so that it is in accordance with orders and then implement them.” (P13, bachelor’s degree, clinical nurse) “I referred the wrong patient for ultrasound, then I realized that I made a mistake, I quickly referred the original patient for ultrasound, but I did not document it.” (P3, bachelor’s degree, clinical nurse). “At the end of the morning shift, I realized that I did not change the patient’s dressing. I asked my evening shift colleague to secretly change it and mark the morning shift on the label.” (P19, bachelor’s degree, clinical nurse).	Failure to record improper performance due to the fear of reprimand. Failure to inform others about an error in implementing the order due to the fear of legal trouble. Concealment of error in the order implementation due to the supervisor’s revealing behavior. The cooperation of nurses in hiding errors to prevent legal trouble for all. Pressure by the head nurse to complete the recording to avoid legal trouble. Documenting the report of the implementation of orders by another nurse. Recording before action. Follow up on the implementation of the forgotten order without documentation. Error correction by colleagues without documentation.	Hiding incorrect implementation of orders. Untruth documentation and separation of the registrar from the agent. Pursuing error correction without documentation.	Unprofessional documentation

### Ethical considerations

The research protocol received approval from the research and ethics committee of the Faculty of Medical Sciences at Tarbiat Modares University (IR.MODARES.REC.1399.232). Participants were provided with comprehensive information about the research objectives and methodology, with the assurance that they could withdraw from the study at any point. Participation was entirely voluntary, and ethical considerations, such as honesty in result presentation, confidentiality of data, and ensuring participant anonymity, were diligently adhered to throughout the study.

## Results

Data analysis led to the development of six main categories, including ‘accuracy in controlling documented orders’, ‘selective and tasteful implementation of orders’, ‘unprofessional documentation’, ‘physical and psychological damage’, ‘instability in managerial interactions’, and ‘unfavorable professional interaction between the doctor and the nurse.’ The brief description of the categories and the theme has been presented in Table 3.

**Table 3:** Illustration of categories and the theme developed in the study

Meaning unit	Category	Description	Theme
Importance and speed in implementing immediate (STAT) orders. Running orders without rechecking. Custom implementation of pro re nata (PRN) orders. Self-administration of medications. Treatment according to the doctor’s order to maintain legal immunity. Different procedures in obtaining and registering telephone orders. Implementation of orders based on the protocol. Hiding incorrect implementation of orders. Untruth recording and separation of the registrant from the agent. Pursuing error correction without documentation. Rechecking documented orders. Accuracy in controlling orders. Endangering patient safety and quality of care. Nurse’s conscience and psychological damage. The inefficiency of managers’ supervision over the implementation of orders. Punitive treatment of the nurse making a mistake in the process of implementing orders. Conflicts between the doctor and nurse in documenting orders. Denial of verbal orders by the doctor. Failure to accept the nurse’s therapeutic suggestions.	Selective and tasteful implementation of orders. Unprofessional documentation. Accuracy in controlling documented orders. Physical and mental injury. Instability in management interactions. Inappropriate professional interaction between the doctor and the nurse.	A strategic approach where nurses chose some specific aspects of medical orders to implement, exercising personal discretion and clinical judgment based on the context and patient needs. It involved a thoughtful and careful selection of elements within the orders influenced by workload, resources, or the overall patient condition. Improper or inadequate recording of information related to patient care and nursing procedures. It involved incomplete, inaccurate, or inconsistent documentation that failed to meet professional standards, potentially compromising the quality of patient records and overall healthcare practices. Meticulous and precise oversight of recorded medical orders as a thorough and vigilant review of documented instructions to ensure they are correct, complete, and align with the prescribed medical orders. Harm or damage affecting both the physical well-being and mental health of patients and nurses. Lack of consistency and inability to engage nurses within the organization. It involved fluctuations in leadership styles and decision-making processes, leading to a potentially challenging work environment. Interactions that do not conform to established standards of professional conduct in a healthcare setting. It involved disrespect, ineffective communication, or failure to collaborate that undermine the quality of patient care.	Selective and tasteful implementation to avoid legal and organizational accountability

### Main concept

The main concern for the nurses in the process of implementing medical orders was ‘legal threat and job prestige’, which revolved around facing legal threats and potential impacts on job prestige in case of making errors in implementing medical orders. The nurses were aware of the potential legal repercussions, and employed heightened diligence and precision in their duties to mitigate the risk of adverse outcomes on their job prestige.

The nurses typically adhered to a practice of double-checking or verifying doctor’s orders, often in pairs, to prevent any oversight that might lead to legal complications. However, in busy wards where time was constrained, this precautionary measure was sometimes compromised, and nurses resorted to implementing orders based on established routines. Recognizing their accountability for nursing interventions following medical orders, nurses meticulously documented the order implementation process. This documentation was designed to serve as a legal safeguard, ensuring that in the event of a legal challenge, they would not face prosecution. In cases of incorrectly implemented orders, there was a tendency to conceal errors, aiming to maintain positive perceptions from nurse managers and preserve their credibility and job positions. Simultaneously, documentation was strategically carried out to bolster the assertion of complete order implementation.

*“I carefully check orders; during the work shift, I implement orders mentally, when writing the report, I check them again so that I write a report exactly the same so that I don’t forget anything and there is no legal trouble.”* (Participant 3, bachelor’s degree, clinical nurse).

*“I constantly try not to make mistakes in the implementation of orders, so that if a problem arises for the patient during the treatment process and the doctor complains about it, I will not be blamed.”* (P18, master’s degree, clinical nurse).

“*If orders are not rechecked and the report is not completely performed, my salary will be reduced and my reputation will be damaged. Therefore, I check orders carefully and write a complete report according to the orders, though I often do not have time to implement them*.” (P7, bachelor’s degree, clinical nurse).

### Context

Excessive individual workloads, the doctor’s failure to comply with legal instructions when issuing verbal orders, and altering orders without notifying nurses at the interpersonal level, along with nurse managers’ insistence on comprehensive documentation at the organizational level, collectively posed challenges to the credibility of nurses’ job performance.

The nurses, faced with time constraints resulting from heavy workloads, found themselves unable to fully and accurately execute medical orders. As a result, they tended to overlook orders and engage in arbitrary prescription practices. Consequently, persistent worries about potential patient harm, the filing of complaints, and feelings of guilt arose due to the inadequate implementation of medical orders.

*“When the ward is busy, one nurse reads the orders and another nurse implement them. The orders are missed or incorrectly implemented. If a patient dies, all nurses will be prosecuted by the law.”* (P5, master’s degree, clinical nurse).

The doctor’s disregard for legal guidelines regarding verbal orders stemmed from a profit-driven motive to incriminate the nurses. Consequently, the nurses harbored concerns about being left defenseless in a court of law, as they followed orders that appeared to lack the doctor’s approval.

*“Once the doctor denied his order on the phone and there was an argument, and finally my colleague was reprimanded. Whenever I receive an order on the phone, I have concerns that the doctor may not accept and may not sign it.”* (P13, bachelor’s degree, clinical nurse).

“*I have seen many times that my colleagues have given verbal orders, but did not register and sign them later, especially when the patient’s condition became a challenge. They prefer not to take the legal responsibility.*” (P14, Anesthesiologist).

Upon reviewing medical orders, certain doctors documented new instructions without notifying the nurses responsible for their implementation. This lack of communication placed the nurses in a vulnerable position, as they could face condemnation for not fully checking and executing these undisclosed orders. Consequently, the nurses consistently harbored concerns about potential legal repercussions arising from the presence of unimplemented orders.

“*Sometimes the doctor writes orders between the previously checked ones, so that if the patient dies, I would be taken responsible for not following up the orders.”* (P1, bachelor’s degree, clinical nurse).

*“I requested a test for the patient, but I forgot to check the creatinine, I went back and added it among the requested ones, because I didn’t want to write a new order.”* (P 16, Orthopedic surgeon).

Under the oversight of higher authorities, nurse managers enforced the thorough documentation of orders, a practice that clashed with the nurses’ reluctance to record comprehensive documentation. Confrontations marked by strict disciplinary measures, along with diminished efficiency and job benefits, served as reprimands. Consequently, the nurses lived with a constant concern that failure to complete documentation might result in disciplinary actions and jeopardize their job security.

*“If someone doesn’t document the report completely, the head nurse will reprimand him/her. If I do not check and document an order by mistake. It will damage our reputation.”* (P10, bachelor’s degree, clinical nurse).

*“For managers, only complete documentation is important and they have nothing to do with the shortcomings of the order implementation.”* (P13, bachelor’s degree, clinical nurse).

### Process

To safeguard their job prestige and credibility, the nurses employed various coping strategies, such as ‘selective and tasteful implementation of orders,’ ‘careful checking of documented orders’, ‘untruth documentation’ and ‘concealment of events’.

Recognizing the patient’s medical file as crucial evidence in legal proceedings, the nurses exercised special caution in managing medical orders to ensure none were overlooked. This precaution aimed to provide a defense in case of legal issues. Additionally, nurses drew lines between previously checked orders to prevent doctors from adding new instructions without notification. However, it is important to note that these measures did not necessarily guarantee the correct implementation of orders.

*“I check orders and document them very carefully, because they are also checked by managers, and if they are not fulfilled, legal and professional consequences will appear.”* (P4, bachelor’s degree, clinical nurse).

“*… for protection and defending myself, I draw a line between orders so that the doctor cannot add a new order.”* (P17, bachelor’s degree, clinical nurse).

### Untruth documentation

The nurses, aware that registering an action was tantamount to carrying it out and could be illegal, composed reports to absolve themselves from potential charges, even when they hadn’t executed a medical order due to various reasons. These reports were crafted in an inaccurate manner, with instances where the nurse documented actions had not been genuinely implemented.

“*Due to a large number of patients, I do not have time to administer antibiotics on time to them all, but I write in the report that the medications have been administered as prescribed.”* (P9, master’s degree, clinical nurse).

### Concealment of events

When faced with legal threats and concerns about their professional reputation, clinical nurses tended to conceal the truth to the greatest extent possible. Their apprehension centered around potential legal complications in the patient’s case and the prospect of becoming entangled in court proceedings or facing repercussions from superiors. Consequently, they refrained from acknowledging mistakes made during the execution of medical orders, intentionally omitting them from nursing reports, and avoiding honest disclosure of errors. It covered up their negligence to escape from legal problems and maintain their professional reputation.

*“Nurses often hide errors in the implementation of orders and dishonestly document them as if they have been implemented, so that if the patient dies and a complaint is filed, they will not be caught by the law.”* (P11, master’s degree, head nurse).

“*I do not tell anyone about errors in the implementation of orders. Because it will be broadcasted everywhere, and it will bring legal trouble to me.”* (P8, bachelor’s degree, clinical nurse).

### Outcomes of the process

Psychological distress among the nurses and reduction of the quality and safety of care were the outcome. Concealment of events and untruth documentation in an effort to perform selective and tasteful interventions to avoid legal and organizational accountability led to patient harm and decreased care quality. Failure to implement medical orders, inaccurate documentation, and concealment of errors harmed patients.

“*I was sleepy during the night shift and I gave 14 units of insulin to the patient by mistake. He suffered from hypoglycemia, but I didn’t tell anyone, and I gave the patient a sugar syrup. It went well. "* (P8, bachelor’s degree, clinical nurse).

The use of unprofessional strategies such as deliberate concealment of events and untruth documentation led to avoiding legal and organizational accountability, but they caused the feeling of guilt, being worried about patient harm, fear of disclosure of error and related psychological burden.

*“I mistakenly injected too much epinephrine to the child, but I didn’t write it in my report, because the supervisor would have given me a heavy work shift if he found out about it. The child developed severe tachycardia. I felt guilty and worried that the child would die.”* (P6, bachelor’s degree, clinical nurse).

### Storyline and the theory

The primary concern for nurses during the implementation of medical orders revolves around the apprehension of patient complaints, legal conflicts, and the fear of managerial blame and job loss, collectively known as ‘legal threat and job prestige.’

Factors contributing to this concern include the doctor’s failure to adhere to legal instructions for verbal orders, the doctor unilaterally adding orders without informing nurses, managerial pressure for comprehensive documentation, and the burden of heavy workloads. In response to this threat, nurses employ protective strategies such as untruthful documentation and concealing events.

**Fig. 1:**
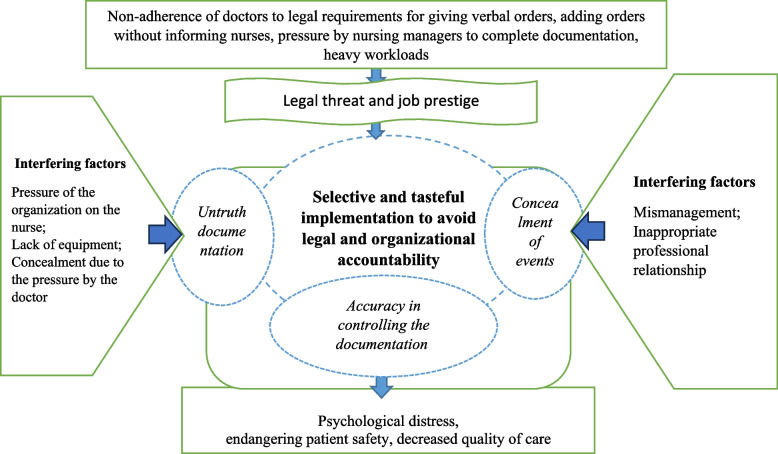
Theory of selective and tasteful implementation to avoid legal and organizational accountability in the process of implementing medical orders by nurses

In the process of implementing orders, the meticulously review doctor’s instructions to prevent unauthorized additions. The demanding workloads prompt nurses to carry out medical orders in a discerning and selective manner. However, in their official reports, used as legal references, they present a different narrative, concealing errors. While this safeguarded them from potential patient complaints and helped maintain their professional reputation, it simultaneously led to patient harm, diminished care quality, and the internal burden of guilt and anxiety about potential error disclosure. These interconnected elements are visually represented in Fig. 1 as the theory of ‘selective and tasteful implementation to avoid legal and organizational accountability.’

## Discussion

This study sought to explore the process of implementing medical orders by clinical nurses working in hospitals in Iran, and identify specific areas of concern in the implementation process, and uncover strategies to address these concerns.

To contextualize our research findings more broadly, we linked our results to the overarching concept of patient safety culture [26]. Accordingly, fostering teamwork within healthcare settings and improving a culture of organizational learning and continuous improvement can address weak dimensions in areas of implementation medical orders. Emphasizing collaboration, communication, and a commitment within the multidisciplinary healthcare team is needed to create an environment conducive to the safe implementation of medical orders by nurses and learning from practice errors.

The nurses had concerns about ‘legal threat and job prestige’ if they made errors in carrying out medical orders. Aware of potential risks, the nurses made concerted efforts to safeguard their professional reputation. The nursing staff implemented certain strategies to protect themselves from adverse legal consequences, resulting in heightened psychological distress and posing a potential threat to patient safety. Nurses play a pivotal role in implementing medical orders, particularly those related to medications, and bear both professional and legal responsibility for ensuring their accurate implementation [2]. The incorrect implementation of medical orders serves as a root cause for legal complications, stress, and anxiety among nurses [9].

Challenges faced by the nurses in performing medical orders encompassed demanding workloads, doctors disregarding legal instructions, altering orders without notifying nurses, and nurse managers exerting pressure for comprehensive documentation, all jeopardizing nurses’ job credibility. The challenges posed by high workloads and fatigue have been identified as significant factors contributing to lapses in implementing medical orders, neglecting the reassessment of orders, and failing to adequately document them [6, 9, 27]. Emphasizing particular aspects of the nursing work environment, including adequate staffing and resources, encouraging nurses’ involvement and career progression are crucial for enhancing the quality of patient care. Hospitals, often regarded as high-risk environments compared to other industries, can benefit significantly from policy measures that prioritize the nursing work environment. By recognizing the importance of this environment in relation to patient safety, policymakers have the potential to mitigate injuries, preserve resources, and cultivate a safety-oriented culture within healthcare settings [28].

The introduction of new orders into the patient’s records by doctors without proper communication to nurses created a sense of threat and anxiety, particularly concerning potential legal conflicts in the future. This issue stemmed from an unprofessional relationship between the doctor and the nurse. According to Schwappach (2016), to ensure completeness and prevent any omissions, it is essential to conduct a thorough review of orders before their implementation [12]. However, in situations of overcrowding, nurses frequently neglect the essential practice of rechecking orders, a lapse that is not only contrary to established protocols but also a violation of legal standards [13].

There is a pressing requirement to enhance professional communication between doctors and nurses [2]. The lack of attention from doctors to nurses’ input during the prescription of medical orders, coupled with a failure to address nurses’ uncertainties about orders, contributes to deficiencies in order implementation [29]. This not only increases the likelihood of errors in carrying out medication orders [17], but also leads to underreporting of errors [8, 30, 31]. Recognizing nurses as active members of the healthcare team and encouraging their active participation in patient care is essential [32].

The refusal of verbal orders by the doctor, coupled with their non-adherence to established rules and legal directives, instilled a sense of legal jeopardy among the nurses. Addressing this issue with verbal orders necessitates the enhancement and regular updating of guidelines and policies. Moreover, the development of policies and instructions should be approached within the framework of multi-professional healthcare [13]. Ethics and ethical considerations as the pivotal elements of patient safety should be included in initiatives for the improvement of the quality and safety of care [33]. Departing from guidelines frequently leads to practice errors. Therefore, a clear understanding of the significance behind adhering to guidelines to prevent errors should be created. Mitigating communication and collaboration barriers, enhancing interprofessional communication, standardizing tasks through protocol implementation, clarifying roles, and fostering teamwork can effectively decrease the occurrence of patient safety incidents [34, 35].

The nurses resorted to tactics such as selectively executing orders, meticulously scrutinizing documented instructions, presenting inaccurate documentation, and concealing events. These measures induced psychological distress and jeopardized the quality and safety of patient care, ultimately leading to harm. The nurses refrained from disclosing events and reporting errors due to apprehensions about legal and professional repercussions. The fear of facing legal consequences, potential job loss, punishment, being subject to blame, and a perceived lack of support from nurse managers act as significant barriers to reporting mistakes [36, 37]. The primary deterrent to error reporting among nurses is the desire to avoid stigmatization, hindering the proactive prevention of future errors [3, 8, 30, 31]. It is imperative for managers to provide support to nurses and establish an effective communication system for error reporting within hospitals [10, 36].

Nurses often underwent psychological strain as a result of incorrectly implementing orders but chose not to disclose their errors. It is a prevailing tendency among nurses to attempt to resolve issues independently and refrain from seeking support [38]. The incidence of errors not only induces stress and moral dilemmas for nurses [8, 30] but also introduces complications for the patient and jeopardizes both patient safety and the overall quality of care [31, 39].

This research revealed that nurses occasionally concealed interventions they had carried out, and in some instances, documented interventions that were not performed. This manipulation of reports by nurses is often driven by the intention to preserve their own and their organization’s credibility. However, it is essential to acknowledge that honesty in documentation is pivotal for preventing future errors [9]. Effective leadership plays a crucial role in supporting nurses to openly express errors [40]. The act of expressing errors is instrumental in mitigating their consequences and preventing their recurrence. Nurse managers should actively work towards modifying the organizational structure and devising plans to foster a positive environment, ultimately reducing the incidence of nursing errors [8].

Consequently, it is crucial for nurse managers to concentrate on empowering nurses to cultivate a culture of accountability and enhance reporting effectiveness. Furthermore, policymakers should prioritize updating nursing education standards to address the imperative issue of patient safety [41, 42].

Given the sensitive nature of the research topic, participants may have felt compelled to offer socially desirable responses or withhold certain information due to concerns about potential repercussions, such as negative judgments or professional consequences, leading to potential underreporting. There’s a possibility that participants may have chosen to conceal crucial data to safeguard their job security. To address this, efforts were made to foster a supportive and non-judgmental environment, establishing rapport and trust with participants. Emphasizing confidentiality and clarifying that the study aimed to comprehend their experiences rather than evaluate their actions were key components in building this trust. Also, the absence of access to non-verbal cues from participants due to the telephone interviews was another limitation of this study.

## Conclusion

The theory of ‘selective and tasteful implementation to avoid legal and organizational accountability’ offers a fresh perspective on the intricate dynamics of how nurses carry out medical orders and what factors influence it. The nurses faced legal threats and potential damage to job prestige if they made errors in carrying out medical orders. Despite being aware of the risks, they worked diligently to minimize the impact on their professional reputation. Challenges included heavy workloads, doctors not following legal instructions, changing orders without informing nurses, and pressure from nurse managers for thorough documentation, all posing threats to job credibility. In response, nurses employed strategies like selectively implementing orders, carefully checking documented orders, providing untrue documentation, and concealing events. Unfortunately, some tactics caused psychological distress and compromised the quality and safety of patient care, ultimately resulting in harm to patients.

Our study findings emphasizes the significance of appropriate strategies to manage legal repercussions and organizational scrutiny. Ineffectual approaches employed by nurses not only jeopardize patient safety but also compromise the overall quality of care, contributing to heightened psychological distress among nurses.

The implications of this study extend beyond its specific context, emphasizing the need for comprehensive planning and policymaking within the healthcare system. It can contribute to global practices in healthcare by shedding light on the underlying factors influencing errors in the implementation of medical orders.

Addressing nursing shortages, enhancing the professional relationship between doctors and nurses, legally protecting nursing professionals, implementing measures such as recording phone communications to prevent order denials, and fostering a culture of honesty in order implementation and documentation are imperative steps for systemic improvement.

Organizations that acknowledge nurses’ concerns as valid, recognizing mistakes as part of the learning process, can play a pivotal role in reducing legal threats and improving the career credibility of nursing professionals, leading to the enhancement in the medical order implementation process. By adopting these measures, organizations can not only elevate the quality and safety of medical order implementation but also contribute to a positive and collaborative work environment.

The emphasis on continued research in this area is crucial for refining strategies and ensuring their applicability across diverse healthcare settings in various healthcare contexts, thus promoting generalizability and widespread positive impact. As the healthcare landscape evolves, these recommendations offer a foundation for systemic improvements that can benefit nursing professionals, patients, and healthcare organizations on a broader scale. To enhance the transferability of findings, it is recommended that further studies be conducted in diverse contexts, allowing for a broader understanding of the issues at hand. The suggested areas of future studies are an extensive literature review to identify existing practical frameworks related to nurses’ implementation of medical orders, legal accountability, and organizational scrutiny in other cultural contexts. Also, research on the impact of various improving strategies for implementing medical orders in the multidisciplinary healthcare context on patient safety and quality of care is required.

### Supplementary Information

**Additional file 1.**

## Data Availability

Due to the qualitative nature of the data collection, and in consideration of the anonymity and confidentiality of the participants, access to the dataset utilized in this research is restricted.
