# Supplementary material for: A grounded theory of the implementation of medical orders by clinical nurses
Source: BMC Nurs. 2024 Feb 13;23:113. doi: 10.1186/s12912-024-01775-6 (PMC10863222; doi:10.1186/s12912-024-01775-6)
Supplement: Supplementary file 1 — Additional file 1. [file 12912_2024_1775_MOESM1_ESM.docx]

Supplementary file 1. The list of questions asked during the interviews

**Demographic data**

- Gender
- Education level
- Nursing work experience

**The open-ended question as the warm up phase**

- How do you implement medical orders in your everyday practice in the ward?

**Probing questions**

- What do you mean?
- Can you please explain it more?
- What was the result of this action?

**Interview questions grounded in theoretical sampling**

- How do you receive, implement, and document telephone orders?
- What new orders were added by the doctor to previously checked ones?
- How do you manage them?
- Did you have any experience with not accepting the nurse's documented phone order from the doctor?
- How did you deal with it and what was the result?
